# Effectiveness of early Anakinra on cardiac function in children with multisystem inflammatory syndrome of COVID-19: a systematic review

**DOI:** 10.1186/s12879-024-09581-w

**Published:** 2024-08-21

**Authors:** Muhammed Shabil, Mahalaqua Nazli Khatib, Godfrey T Banda, Quazi Syed Zahiruddin, Suhas Ballal, Pooja Bansal, Manish Srivastava, Isha Arora, M Ravi Kumar, Aashna Sinha, Kumud Pant, Jumana M. Al-Jishi, Hawra Albayat, Mona A. Al Fares, Mohammed Garout, Hayam A Alrasheed, Maha F. Al-Subaie, Ali A. Rabaan

**Affiliations:** 1 Center for Global Health Research, Saveetha Institute of Medical and Technical Sciences, Saveetha Medical College and Hospital, Saveetha University, Chennai, India; 2 Medical Laboratories Techniques Department, AL-Mustaqbal University, Hillah, Babil, 51001 Iraq; 3 Division of Evidence Synthesis, Global Consortium of Public Health and Research, Datta Meghe Institute of Higher Education, Wardha, India; 4 School of Global and Public Health, Kamuzu University of Health Sciences, Blantyre, Malawi; 5 South Asia Infant Feeding Research Network (SAIFRN), Division of Evidence Synthesis, Global Consortium of Public Health and Research, Datta Meghe Institute of Higher Education, Wardha, India; 6 Department of Chemistry and Biochemistry, School of Sciences, JAIN (Deemed to be University), Bangalore, Karnataka India; 7 Department of Allied Healthcare and Sciences, Vivekananda Global University, Jaipur, Rajasthan, 303012 India; 8 Department of Cardiology, NIMS University, Jaipur, India; 9 Chandigarh Pharmacy College, Chandigarh Group of College, Jhanjeri, Mohali, 140307 Punjab India; 10 Department of Chemistry, Raghu Engineering College, Visakhapatnam, 531162 Andhra Pradesh India; 11 School of Applied and Life Sciences, Division of Research and Innovation, Uttaranchal University, Dehradun, India; 12 Department of Biotechnology, Graphic Era (Deemed to be University, Clement Town Dehradun, Dehradun, 248002 India; 13 Department of Allied Sciences, Graphic Era Hill University Clement Town Dehradun, Dehradun, 248002 India; 14 Internal medicine department, Qatif central hospital, Qatif, 32654 Saudi Arabia; 15 Infectious Disease Department, King Saud Medical City, Riyadh, 7790 Saudi Arabia; 16 Department of Internal Medicine, King Abdulaziz University Hospital, Jeddah, 21589 Saudi Arabia; 17 Department of Community Medicine and Health Care for Pilgrims, Faculty of Medicine, Umm Al-Qura University, Makkah, 21955 Saudi Arabia; 18 Department of Pharmacy Practice, College of Pharmacy, Princess Nourah bint Abdulrahman University, Riyadh, 11671 Saudi Arabia; 19 Research Center, Dr. Sulaiman Alhabib Medical Group, Riyadh, 13328 Saudi Arabia; 20 College of Medicine, Alfaisal University, Riyadh, 11533 Saudi Arabia; 21 Molecular Diagnostic Laboratory, Johns Hopkins Aramco Healthcare, Dhahran, 31311 Saudi Arabia; 22 Department of Public Health and Nutrition, The University of Haripur, Haripur, 22610 Pakistan

**Keywords:** Anakinra, Multisystem inflammatory syndrome in children, Good health and well-being, COVID-19, Systematic review

## Abstract

**Background:**

Multisystem Inflammatory Syndrome in Children (MIS-C) associated with SARS-CoV-2 can lead to severe cardiovascular complications. Anakinra, an interleukin-1 receptor antagonist, is proposed to benefit the hyperinflammatory state of MIS-C, potentially improving cardiac function. This systematic review evaluated the effectiveness of early Anakinra administration on cardiac outcomes in children with MIS-C.

**Methods:**

A comprehensive search across PubMed, Embase, and Web of Science until March 2024 identified studies using Anakinra to treat MIS-C with reported cardiac outcomes. Observational cohorts and clinical trials were included, with data extraction focusing on cardiac function metrics and inflammatory markers. Study quality was assessed using the Newcastle-Ottawa Scale.

**Results:**

Six studies met the inclusion criteria, ranging from retrospective cohorts to prospective clinical studies, predominantly from the USA. Anakinra dosages ranged from 2.3 to 10 mg/kg based on disease severity. Several studies showed significant improvements in left ventricular ejection fraction and reductions in inflammatory markers like C-reactive protein, suggesting Anakinra’s role in enhancing cardiac function and mitigating inflammation. However, findings on vasoactive support needs were mixed, and some studies did not report significant changes in acute cardiac support requirements.

**Conclusion:**

Early Anakinra administration shows potential for improving cardiac function and reducing inflammation in children with MIS-C, particularly those with severe manifestations. However, the existing evidence is limited by the observational nature of most studies and lacks randomized controlled trials (RCTs). Further high-quality RCTs are necessary to conclusively determine Anakinra’s effectiveness and optimize its use in MIS-C management for better long-term cardiac outcomes and standardized treatment protocols.

**Supplementary Information:**

The online version contains supplementary material available at 10.1186/s12879-024-09581-w.

## Introduction

Multisystem Inflammatory Syndrome in Children (MIS-C) is a rare but severe complication associated with SARS-CoV-2 infection, primarily affecting children and adolescents [1]. This condition, which shares clinical features with Kawasaki disease and toxic shock syndrome, can lead to a hyperinflammatory state and multiorgan dysfunction, including cardiovascular involvement [1, 2]. While the exact pathophysiology of MIS-C remains unclear, dysregulated immune responses and cytokine storms are believed to play a crucial role.

The cardiac manifestations associated with MIS-C highlight the syndrome’s potential to induce severe cardiovascular damage, which can lead to critical outcomes if not addressed promptly [3–5]. Myocardial dysfunction in MIS-C, characterized by reduced left ventricular ejection fraction (LVEF), can compromise cardiac output, leading to shock and necessitating intensive care support [6, 7]. Similarly, coronary artery abnormalities, such as the development of aneurysms, pose a risk for thrombosis, myocardial infarction, and long-term cardiac morbidity, emphasizing the critical need for early diagnostic imaging and continuous monitoring [8, 9]. The implications of these cardiac complications extend beyond the acute phase of MIS-C, with potential long-term sequelae requiring ongoing cardiological follow-up to monitor for and manage any enduring cardiovascular damage [10].

Experiences with other inflammatory conditions, such as Kawasaki disease and cytokine release syndromes, have largely guided MIS-C treatment. Immunomodulatory therapies have been employed to manage MIS-C, including intravenous immunoglobulin (IVIG), corticosteroids, and biologics targeting specific inflammatory pathways [11–13]. Among these therapeutic options, anakinra, an interleukin-1 receptor antagonist, has garnered attention for its potential to mitigate the hyperinflammatory state associated with MIS-C [14–16].

Anakinra, a recombinant form of the naturally occurring interleukin-1 receptor antagonist, has been used to treat various autoinflammatory and rheumatic diseases [17]. The rationale for using anakinra in this context is grounded in its successful application in other inflammatory conditions, such as rheumatoid arthritis and systemic juvenile idiopathic arthritis, where it has been shown to reduce inflammation markers and improve clinical outcomes significantly. By blocking the actions of interleukin-1, a potent pro-inflammatory cytokine implicated in the pathogenesis of MIS-C, anakinra may help reduce systemic inflammation and prevent end-organ damage, including cardiac dysfunction [18, 19]. Early administration of anakinra in children with MIS-C has been proposed as a potential strategy to prevent or mitigate cardiovascular complications. However, the effectiveness of this approach remains uncertain, as the available evidence is limited and derived from case reports, case series, and small observational studies [14, 20, 21].

Given the potential severity of cardiac involvement in MIS-C and the need for timely and effective management, it is crucial to systematically evaluate the existing literature on the efficacy of early anakinra treatment in preserving or improving cardiac function in these pediatric patients. This systematic review aims to synthesize and critically appraise the available evidence from published studies to assess the impact of early anakinra administration on cardiac outcomes, such as left ventricular function, coronary artery abnormalities, and other cardiovascular manifestations in children with MIS-C.

## Methods

The study selection process was documented using the Preferred Reporting Items for Systematic Reviews and Meta-Analyses (PRISMA) flow diagram (Table S1) [22]. The study protocol has been registered with PROSPERO.

### Search strategy

A systematic literature search was conducted in multiple electronic databases, including PubMed, Embase, and Web of Science, from inception to March 05, 2024. The search strategy utilized a combination of relevant medical subject headings (MeSH) and text words related to “multisystem inflammatory syndrome in children,” “MIS-C,” “pediatric inflammatory, multisystem syndrome,” “anakinra,” “interleukin-1 inhibitors,” No language restrictions were applied. The complete search strategy is documented in Table S2.

### Eligibility criteria

Studies were considered eligible for inclusion if they were observational studies (cohort studies, case-control studies, case series) or clinical trials such as randomized controlled trials (RCTs) or quasi-experimental studies reported using anakinra in children diagnosed with MIS-C associated with SARS-CoV-2 infection. The population of interest was children and adolescents aged 21 years or younger with MIS-C. Eligible studies must have involved the administration of anakinra, either as monotherapy or in combination with other treatments, to manage MIS-C. Additionally, studies were required to report on at least one cardiac outcome, such as left ventricular function (e.g., ejection fraction, fractional shortening), or other cardiovascular manifestations (e.g., myocarditis, arrhythmias, shock), or inflammatory biomarkers such as C-reactive protein (CRP). Studies with or without a control group, where patients received standard care, placebo, or other treatments, were considered eligible for inclusion. Case reports, reviews, editorials, comments, and studies that did not report on cardiac outcomes or the use of anakinra were excluded.

### Study selection

Two independent reviewers screened the titles and abstracts of the identified studies for potential eligibility using semiautomated software (Nested-Knowledge, MN, USA) [23]. Full texts of potentially relevant studies were then retrieved and assessed against the predefined inclusion and exclusion criteria. Any disagreements were resolved through discussion or consultation with a third reviewer.

### Data extraction

A standardized data extraction form was used to collect relevant information from the included studies, such as study characteristics (authors, publication year, study design, location, sample size), participant characteristics (age, sex, comorbidities), MIS-C diagnostic criteria, anakinra dosing and timing of administration, change in cardiac-related outcome and parameters. Two reviewers independently extracted the data, and any discrepancies were resolved through discussion or consultation with a third reviewer.

### Quality assessment

The methodological quality of the included studies was assessed using the Newcastle-Ottawa Scale (NOS). Two reviewers independently performed the quality assessment, and any disagreements were resolved through discussion or consultation with a third reviewer.

## Results

### Literature search

**Fig. 1:**
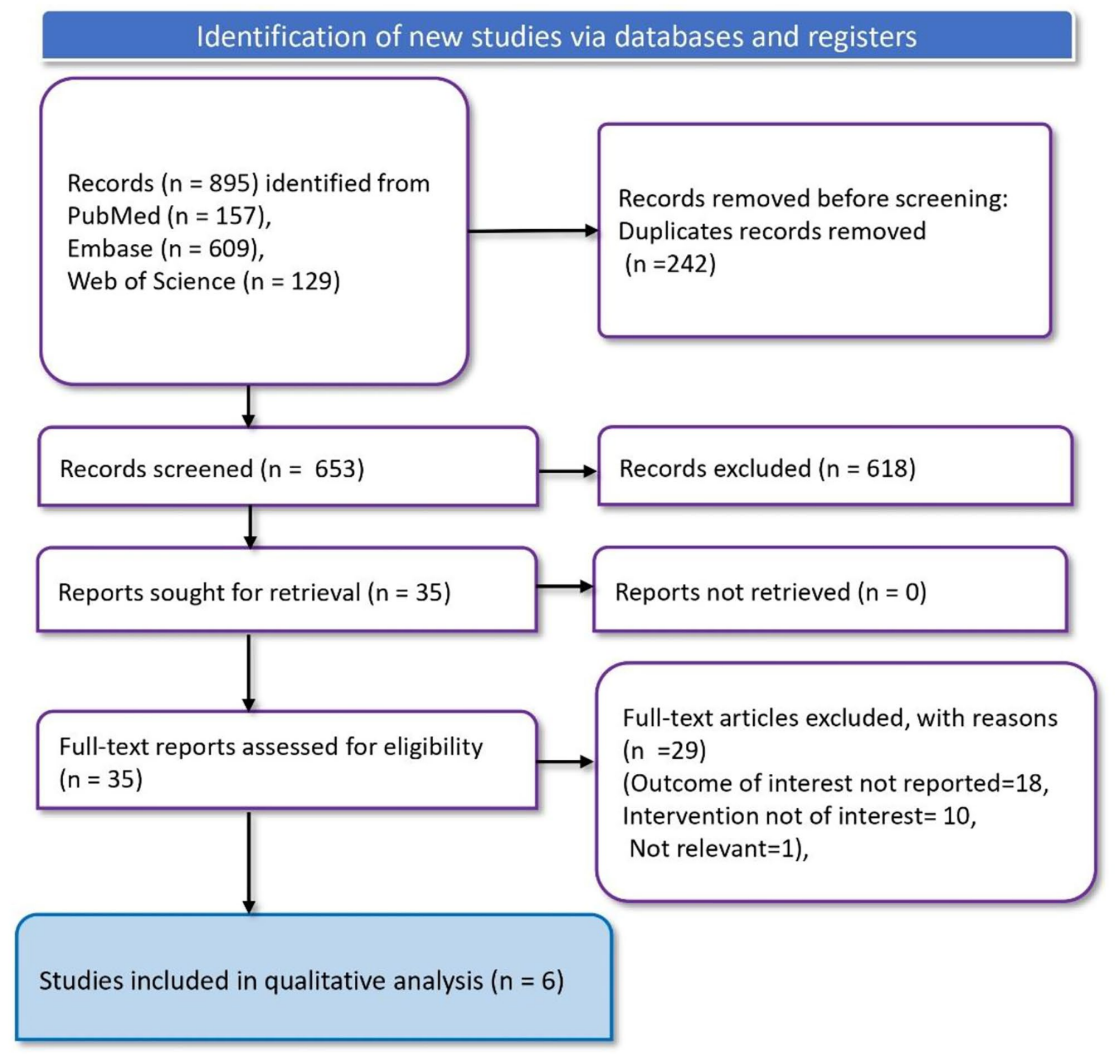
PRISMA flowchart depicting article screening and study selection process

In the literature search, 895 records were identified through various databases: 157 from PubMed, 609 from Embase, and 129 from the Web of Science. From these, 242 duplicate records were removed before screening. Following this removal, 653 records were screened, excluding 618 records. Thirty-five articles were then subjected to a full-text assessment for eligibility. Of these, 29 full-text articles were excluded for various reasons: the outcome of interest was not reported in 18 studies, the intervention was not of interest in 10 studies, and 1 study was deemed irrelevant. Consequently, six studies [24–29]were included in the qualitative analysis (Fig. 1).

### Characteristics of the included studies

The important characteristics of the included studies are presented in Table 1. The research predominantly comes from the USA, with a single study from France and Italy. The designs range from retrospective cohort studies to prospective studies, focusing on pediatric populations diagnosed with MIS-C as defined by the Centers for Disease Control and Prevention (CDC), World Health Organization (WHO) criteria, or the Royal College of Paediatrics and Child Health (RCPCH) criteria in one instance. The number of participants varied, with the smallest study including 14 participants and the largest encompassing 1516. The mean or median ages of subjects typically ranged from 6 to 10 years old, reflecting a young cohort affected by MIS-C. Anakinra dosages and frequencies were diverse; some studies used it alongside intravenous immunoglobulin (IVIG) and glucocorticoids as part of a comprehensive treatment regimen. Specific doses of anakinra varied from 2.3 mg/kg to 10 mg/kg per day, adjusted for disease severity, and administered subcutaneously or intravenously. Quality assessment of the studies are given in Table S3.

**Table 1:** Characteristics of included studies

Author	Year	Country	Study Design	Study Population	Total participants	Mean (SD) or median (IQR) age	MIS diagnosis criteria	Number of patients with anakinra treatment	Dose and frequency of anakinra	Key findings
Akkoyun 2023 [24]	2023	USA	Retrospective cohort study	Children hospitalized for MIS-C	138	9.9 (8.1–13.9) years	CDC criteria	32	IV anakinra was started at 8–10 mg/kg/day divided every 6 h in critically ill patients and 6–8 mg/kg/day divided every 6 h in non-critically ill patients	Anakinra use showed no differences in median duration of vasoactive support, fever resolution, CRP reduction, or Vasopressor-Inotrope Score
Chang 2023 [25]	2023	USA	Retrospective cohort study	MIS-C cases in a US surveillance registry	1516	10.1 (4.1) years	CDC criteria	121	4 mg/kg/day (range 0.2–20 mg/kg/day)	No short-term cardiovascular benefits observed with the early addition of anakinra to IVIG and glucocorticoids compared to using IVIG and glucocorticoids alone.
Dizon 2023 [26]	2023	USA	Observational clinical cohort study	Children hospitalized for MIS-C	46	8 (4–10) years	CDC criteria	32	8–10 mg/kg/day divided every 6 h in critically ill patients and 6–8 mg/kg/day divided every 6 h in non-critically ill patients	Anakinra treatment, primarily used alongside IVIG in patients with severe MIS-C, was linked to enhanced cardiac function and fever reduction
Dusser 2024 [27]	2024	France	Prospective study	Children Hospitalized for MIS-C	470	10.06 (3.9) years	CDC criteria	18	2.53 ± 1.3 mg/kg/day for a median duration of 3 days	Subcutaneous anakinra is a safe and effective option for treating heart failure or MAS in MIS-C.
Taddio 2024 [28]	2024	Italy	Retrospective cohort study	Children with MIS-C	239	8 (I4–11) years	RCPCH criteria	21	NA	Early treatment with anakinra is both safe and highly effective in patients with severe MIS-C.
Yang 2023 [29]	2023	USA	Retrospective cohort study	Hospitalized pediatric patients (age ≤ 21 years) for MIS-C	14	6 (4.0-10.5) years	NA	14	Initial median dose of 2.3 mg/kg with a median dosing interval of 12 h for a median initial treatment duration of 3.5 days	Clinicians should assess patient history and previous therapy responses before starting IV anakinra. Further extensive studies are needed to confirm its off-label safety and efficacy.

Abbreviations: CDC: Centers for Disease Control and Prevention, CRP: C-reactive protein, IV: Intravenous, IVIG: Intravenous Immunoglobulin, IQR: Interquartile Range, MAS: Macrophage Activation Syndrome, MIS-C: Multisystem Inflammatory Syndrome in Children, RCPCH: Royal College of Paediatrics and Child Health, SD: Standard Deviation, VIS: Vasopressor-Inotrope Score

### Left ventricular ejection fraction (LVEF)

Chang et al. [25] observed that the time to normalize LVEF and the prevalence of left ventricular dysfunction did not significantly differ between the Anakinra and control groups on days 3–4. In contrast, Dizon et al. [26] documented that patient in the Anakinra group, particularly those with initially low LVEF or those receiving combined therapy (IVIG, Anakinra, and corticosteroids), exhibited significant improvements in LVEF. Dusser et al. supported these findings, noting a rapid enhancement in heart function across all patients, with LVEF values rising above 55% shortly after treatment initiation.

### Biomarkers

Significant reductions in inflammatory markers such as CRP, ferritin, and D-dimer were reported by Yang et al. [29], where median levels showed substantial decreases from baseline to the final dose of Anakinra. This was accompanied by a decrease in nearly all patients’ marker levels, demonstrating a strong anti-inflammatory response. Additionally, Dusser et al. [27] observed that CRP levels dramatically reduced from an average of 146 mg/L to 43 mg/L within approximately 2.8 days, reinforcing the rapid anti-inflammatory effects of Anakinra.

### Cardiac presentation at discharge

Taddio et al. [28] highlighted that Anakinra treatment was associated with a lower prevalence of heart involvement at discharge (3 out of 21 patients), compared to those who did not receive Anakinra (66 out of 169 patients). This finding suggests a protective role of Anakinra against end-stage cardiac complications in MIS-C.

### Overall cardiac function

An overall improvement in cardiac function was evident in findings from Dizon et al. [26] and Dusser et al. [27], where Anakinra was beneficial across various cardiac function metrics, including LVEF and cardiac strain, especially in patients presenting with severe clinical features. The improvement in cardiac biomarkers, as observed in the studies by Yang et al. [29] and Dusser et al. [27], further underscores the beneficial impact of Anakinra on overall cardiac health in MIS-C patients.

### Vasoactive support and vasoactive inotrope score (VIS)

Akkoyun et al. [24]noted no significant differences in the need for vasoactive support or inotrope scores between the Anakinra-treated and control groups. Interestingly, a higher incidence of new or worsened left ventricular dysfunction was seen in the Anakinra group, although this was not statistically significant. This suggests a complex interaction between Anakinra treatment and severe cardiac manifestations. Furthermore, Chang et al. [25] reported that Anakinra addition to standard therapy did not significantly alter the need for vasopressors, indicating limited efficacy in reducing acute cardiac support needs.

These findings collectively illustrate that while Anakinra may offer considerable benefits in mitigating cardiac involvement at discharge and systemic inflammation, its effects on acute cardiac support and overall left ventricular function are more nuanced. These effects likely depend heavily on the patient’s initial cardiac condition and the severity of their inflammatory symptoms.

## Discussion

This systematic review critically evaluates the effects of early administration of Anakinra on cardiac outcomes in children with MIS-C, a severe complication related to SARS-CoV-2 infection. The studies incorporated data spanning observational cohorts, providing a mixed results regarding the effectiveness of anakinra on cardiac parameters.

Anakinra, a recombinant interleukin-1 receptor antagonist, demonstrates potential in mitigating severe inflammatory reactions in MIS-C, similar to its effects in other cytokine-mediated diseases. This systematic review finds that Anakinra may positively influence cardiac function, evidenced by significant improvements in LVEF and reductions in inflammatory markers such as CRP, ferritin, and D-dimer, particularly in patients with compromised myocardial function at baseline. However, the therapeutic outcomes vary. For instance, Chang et al. [25] observed no significant changes in LVEF or reductions in vasopressor needs, suggesting that the effectiveness of Anakinra might depend on the timing of administration, the severity of the disease at onset, and specific patient characteristics. Despite these variances, Taddio et al. reported a lower prevalence of cardiac involvement at discharge in Anakinra-treated patients, indicating a protective effect against acute cardiac complications and possibly reducing long-term cardiac morbidity. The mixed findings on the need for vasoactive support and observed incidences of LVEF suggest a complex interplay between Anakinra treatment and cardiac function, which calls for further investigation into the optimal dosing and administration timing to maximize its benefits. The available evidence is very limited for reaching any strong conclusion in the effectiveness of Anakinra in improving cardiac outcome in MIS-C.

A previous review assessed the treatment options for MIS-C [13]. IVIG and glucocorticoids are the cornerstones of treatment, reflecting their efficacy in similar inflammatory conditions like Kawasaki Disease (KD). IVIG is often used as the first-line treatment due to its multiple anti-inflammatory mechanisms, which include modulation of immune cell function and suppression of cytokine production. Glucocorticoids are utilized for their potent anti-inflammatory properties, particularly in severe cases where rapid modulation of the immune response is necessary to prevent organ damage. Biologic agents such as anakinra and tocilizumab are reserved for more severe or refractory cases. They authors mentioned that Anakinra can be beneficial for cases where standard treatments fail, helping to mitigate the hyperinflammatory state by blocking a key pathway in the inflammatory process. It is particularly effective in critically ill patients or those not responding adequately to IVIG and glucocorticoids, quickly reducing inflammation and improving clinical outcomes, especially in severe disease manifestations that involve significant cardiac complications [13]. Despite the application of these therapies, the treatment of MIS-C remains challenging due to the variability in disease severity and response to treatment. The effectiveness of these modalities is continually being evaluated, highlighting the need for more targeted research to optimize therapeutic strategies and improve outcomes in MIS-C patients. The review emphasizes the limited evidence and the need for RCTs to provide more definitive guidance on managing this complex condition.

The use of Anakinra should be considered carefully, weighing its benefits against potential risks and the effectiveness of other treatments. The decision to use Anakinra may benefit from being tailored to individual patient profiles, particularly considering disease severity and initial response to standard therapies. Institutions may need to develop protocols that allow for the flexible integration of Anakinra based on real-time clinical assessments and evolving patient needs. For MIS-C, the National Institutes of Health (NIH) recommends considering additional immunomodulatory therapies [30]. These include high-dose anakinra, higher-dose glucocorticoids, or infliximab, all categorized as BIIb recommendations due to limited evidence on the most effective agent for intensification therapy in this group. In severe cases, combination therapy may involve higher-dose glucocorticoids with either anakinra (BIII) or infliximab (BIII), but anakinra and infliximab should not be used together. The panel advises against a second dose of IVIG for intensification therapy due to its common resistance, the rapid progression of the disease, and the potential risks of fluid overload in MIS-C patients. Anakinra, preferred for its short half-life (4–6 h) allowing for rapid discontinuation, is recommended at high doses (5–10 mg/kg/day). This recommendation is based on its proven effectiveness in macrophage activation syndrome and its use as a steroid-sparing agent in MIS-C management, potentially extending up to two weeks [30]. Further research is needed to define the optimal role of Anakinra in MIS-C treatment protocols. Prospective RCTs and larger observational studies should aim to clarify the conditions under which Anakinra is most effective. Additionally, studies should explore the pathophysiological underpinnings of MIS-C to better understand the inflammatory mechanisms at play and identify the most appropriate therapeutic targets. Most studies focus on short-term outcomes without addressing long-term cardiac health. Given the potential for chronic cardiovascular sequelae in MIS-C, longitudinal studies are necessary to understand the full spectrum of Anakinra’s benefits.

Our study has some limitations. The included studies predominantly utilized observational designs, which inherently carry a higher risk of bias compared to RCTs. The variability in study design and the observational nature of the data may influence the reliability and applicability of the findings. The cardiac outcomes reported across the studies varied significantly. This heterogeneity in outcome measures precluded the possibility of conducting a meta-analysis, which would have provided a more comprehensive and quantitative synthesis of the data. The absence of uniform outcome metrics across the studies complicates the interpretation of Anakinra’s overall effectiveness on cardiac functions in MIS-C patients. Several studies lacked a control group, which is crucial for establishing comparative baselines and understanding the true effect of Anakinra independent of external variables. The absence of control groups in these studies significantly limits the ability to attribute observed improvements directly to Anakinra treatment. Another critical limitation is the small number of studies that specifically reported cardiac-related outcomes associated with Anakinra in the context of MIS-C. The limited data availability restricts our ability to draw broad conclusions and diminishes the strength of the evidence supporting Anakinra’s efficacy. The studies included in this review did not adequately account for potential confounders and biases, which could skew results and lead to misleading interpretations. This lack of adjustment for confounding variables undermines the validity of the findings and suggests that the observed effects might not solely be attributable to Anakinra therapy.

While this review provides valuable insights into the potential benefits of Anakinra for treating cardiac complications in MIS-C, the evidence is weakened by significant methodological limitations. There is a pressing need for more high-quality, RCTs that are adequately powered, include control groups, and standardize cardiac outcome measures to definitively ascertain the effectiveness of Anakinra in this clinical setting. Such studies would help clarify the role of Anakinra in MIS-C treatment protocols and potentially guide clinical practice more effectively.

## Conclusion

Early administration of Anakinra in children with MIS-C may be beneficial for improving cardiac function and reducing systemic inflammation. However, the current evidence is insufficient to conclude and make any clinical recommendation regarding the effects of Anakinra on cardiac function in MIS-C. Further research is needed to refine the therapeutic strategies involving Anakinra, including more rigorous RCTs and studies focusing on long-term outcomes to fully ascertain its role in the management of MIS-C.

### Electronic supplementary material

Below is the link to the electronic supplementary material.

Supplementary Material 1

## Data Availability

All data are presented within the manuscript and are available by contacting the corresponding author.
